# Competition among health care providers: helpful or harmful?

**DOI:** 10.1007/s10198-015-0736-3

**Published:** 2015-10-14

**Authors:** Pedro Pita Barros, Werner B. F. Brouwer, Sarah Thomson, Marco Varkevisser

**Affiliations:** Universidade Nova de Lisboa, Lisbon, Portugal; Erasmus University Rotterdam, Rotterdam, The Netherlands; WHO Barcelona Office for Health Systems Strengthening, Barcelona, Spain

## Introduction

The idea of competition in health care can provoke strong reactions from commentators, with some for and others against. Proponents of competition generally fall into one of two camps: those who believe in the innate value of market-based resource allocation (i.e., a decentralised approach to production and consumption decisions, with prices providing the main signals for such decisions) and those who favour the market more for its potential to correct the failures of government regulation. Both camps typically expect competition to do the following things: strengthen patient choice, stimulate innovation, improve quality, enhance efficiency and control costs — in short, to give people what they want in the least costly way possible. Opponents of competition, in contrast, typically fear it will lead to undesirable outcomes such as a reduction in quality, access to health care based on ability to pay rather than medical need and, as a result, inequity and inefficiency in the distribution of health services.

Given these differing views, the European Commission asked its Expert Panel on Effective Ways of Investing in Health to consider if and how competition among health care providers might benefit health systems in the European Union. This article summarises the main findings and conclusions of the panel’s final report [8].^1^

## Instrument rather than goal

Competition is an instrument for organising decisions about the use of resources.^2^ Its primary purpose is to improve efficiency (value for money). In the context of health systems, improving efficiency is important in so far as it contributes to the attainment of broader goals such as ensuring all people are able to use needed, cost-effective, quality health services without experiencing financial hardship. Neither competition nor strict reliance on government regulation is likely to be effective in achieving all of these goals. Attempts to avoid or correct market failure can result in government failure, and vice versa.

## One size does not fit all

Providers usually compete for patients, based on price or quality or both price and quality, but they may also compete for budgets — for example, in the case of auctions for public–private partnership contracts to provide a specific service or through choice of location (which may either help to attract or deter patients). Each of the three broad types of competition has its own set of requirements, as set out in Table 1. Which type is most suitable will depend on the characteristics (structure) of a given health care market.

**Table 1:** Types of provider competition

Type of competition	Requirements for competition	Information needs
Competition among health care providers in a market	Several providers; goods and services more or less substitutable; providers have freedom over relevant aspects of the service they provide; patients have free choice of provider; providers compete to attract patients; payment to providers based on patients treated	Information about location, quality and prices of providers; information available to patients and to third-party payers
Competition for the market (e.g., competitive bidding)	Several providers (fewer than above) compete for the right to provide a service or goods; only one or a few providers will be selected in a geographical area or to provide a specific service (e.g., drugs, public–private partnerships for hospitals); mechanisms to determine the selected provider include tenders and auctions	Ability to describe the service or goods in an accurate and verifiable way
Yardstick competition	Provider incentives based on comparative performance information; more useful when providers are geographically dispersed and not directly competing with rivals	Good information about providers; definition of performance indicators

The conditions required for provider competition to improve health system performance vary across countries, health system sub-sectors and time. They include: adequate information about provider prices and quality and the ability of patients to interpret this information; standardised products and services; the existence of multiple providers and buyers; and easy entry and exit for providers. Information about quality is especially important if competition (and patient choice) are intended to improve access to high-quality care. At least in theory, medical goods, such as equipment and pharmaceuticals, tend to meet the conditions for effective competition. For clinical services the introduction or expansion of competition present considerable difficulties, in particular because of the presence of information asymmetries.

Policies to introduce or increase competition should be informed by analysis of the conditions in place and probable effects. They should also be accompanied by policies that allow the market to function in line with predefined objectives.

## Provider competition and patient choice

Provider competition is not the same as patient choice of provider. Both concepts emerged at around the same time in many national health policy debates. Because of this, they are often seen as two sides of the same coin, but they are in fact distinct. Though not uncontroversial, patient choice is an important principle in some health systems. It does not, however, automatically imply competition among health care providers. Health systems can provide patients with alternatives to choose from even if providers do not compete for patients. For example, providers may compete to be included in a network of providers, without patients having choice of provider, or health professionals may choose among competing providers on behalf of patients. Patient choice is thus possible without provider competition, and vice versa.

## What does economic theory say about provider competition?

Information on relevance, quality, process and outcome is critical if competition is to enhance efficiency in health care. Where there is adequate information about quality of care and dominant positions are absent, economic theory suggests competition will force organisations to be more efficient and innovative and may therefore reduce unit prices. If prices are regulated and quality is observable as well as used to guide demand, economic theory predicts competition to improve health service quality.^3^ If prices are not regulated but set by providers themselves, however, there is no theoretical presumption about the impact of competition on quality.

The asymmetry of information that characterises much health care creates considerable scope for supplier-induced demand. Competition can therefore increase the number of services provided and billed, thereby increasing overall health system expenditure. This higher spending may or may not be justified by additional health benefits for (some parts of) the population.

Concerning geographical access to health services, more competition can improve this dimension of quality by encouraging the entry of new providers. It may prove harmful, however, where a low population density, and thus aggregate demand, makes the provision of some health services economically non-viable without subsidies, or where there are inadequate measures to prevent the emergence of local/regional monopolies for the provision of health services that require a minimum efficient scale.

By accommodating the heterogeneity of patients in a decentralised manner, competition may also contribute to a more responsive health system.

## What does the (limited) empirical evidence tell us?

The empirical evidence base on competition among providers of health care is small but expanding. It focuses on a limited set of countries. For competition among hospitals, most studies are from the USA [9] and the UK [5, 11], while for competition in primary care the Nordic countries are most often studied.^4^ In the market for (generic) medicines, many European countries—including, for example, the Netherlands [1]—use tendering as an instrument to lower spending on outpatient prescription pharmaceuticals through competitive bidding and other negotiation mechanisms to provide wider access at lower prices [6].

Under regulated prices, the studies reviewed by Gaynor and Town [10] point to a positive impact of competition on quality of care (proxied by mortality rates), but these studies are performed in the context of USA and UK health systems only. Under market-determined prices, the empirical evidence shows a more diverse picture with an almost equal occurrence of positive, zero and negative effects of competition on quality. Concerning the empirical evidence on competition in hospital markets, the heavy reliance in the literature on hospital mortality rates (almost the sole indicator used to measure the effects of competition on performance) is very controversial, given its major limitations.

Competition may also have an effect on equity of access to health care. The limited evidence, however, does not allow for general presumptions about the effects of competition on equity of access to health care.^5^

## Know your market

The impact of competition on the health system, on the demand and supply of health care and, ultimately, on the health of the population is highly conditional on the environment in which it is introduced. Empirical evidence reflects the specificities of context, with results varying over time and by market, country and policy design. Apparently minor differences in market characteristics can lead to very different outcomes. Fostering more competition in markets in which providers already compete effectively is unlikely to result in significant additional benefit.

## Anticipate unintended consequences

Provider competition may have unanticipated adverse effects, especially if introduced without careful analysis of the relevant market and context or without ensuring the necessary conditions are in place. Competing providers may focus on quality indicators that are being measured while at the same time neglecting (important) aspects of quality that are not being measured. If competition lowers prices, there may be a strong volume effect (an increase in the use of services) and expenditure may rise. A decrease in competition leading to higher prices can also trigger a positive volume response if providers are able to induce demand, increasing expenditure. The social value of higher volume and expenditure may well depend on underlying causes. Policy makers need to anticipate how competition is likely to unfold, monitor early effects and be prepared to act swiftly to address adverse developments.

## Provider competition can improve health system performance, but it’s not a panacea

Provider competition can contribute to stronger performance, but it will not address all performance problems and may have adverse effects. Neither economic theory nor empirical evidence support the conclusion that competition should be promoted for all health services. As a result, policy makers need to think carefully about where, when and how to introduce or increase competition. They also need to be ready to respond to unintended consequences.

## The devil is in the detail

The panel’s report concludes that competition can improve access to health care; it may help to achieve lower unit costs at the micro level, although aggregate costs will often increase at the macro level; it may improve quality of care if information about quality is reliable and pertinent and prices are regulated; the extremely limited existing evidence says little about its impact on equity. It is critical to note, however, that it is not possible to make general presumptions about the effects of provider competition in practice. Actual effects depend, to a very significant extent, on the specific context: the devil is in the detail.

## Implications for policy

When considering the use of competition among health care providers, policymakers should take into account the following:

1. Introducing or increasing competition in the provision of health care is a challenging undertaking. The conditions for success and risks for failure need to be carefully assessed in every case. In the right context, and with appropriate policy design, introducing competition can help to meet some health system objectives, although it is unlikely to contribute simultaneously and positively to all.
2. The introduction of provider competition requires additional policy actions—first, to ensure that the market functions properly; and second, to ensure there is careful, constant evaluation of outcomes. Ensuring market transparency through the availability of information on quality, price and other relevant dimensions, to the extent that this is feasible and affordable, is essential if competition is to improve health system performance. However, the challenge of measuring and comparing quality across services should not be underestimated.
3. The introduction of provider competition also requires, among other things, the enforcement of competition rules to prevent the creation, strengthening and abuse of dominant positions.
4. Policy concerns about equity underline the need for careful monitoring of how the introduction of (or an increase in) provider competition affects different groups of people. While there is no general presumption about the impact of competition on equity, competition is not, in general, the best instrument for addressing equity concerns. Effects on equity need to be considered on a case by case basis—before to predict, and after to monitor results.
5. Finally, given the paucity of empirical evidence, new models based on competition should incorporate a rigorous evaluation to ensure that the desired effects are being obtained and to inform future policies. Empirical analysis must, however, be interpreted in the light of the specific context: it does not produce universal rules. Health systems are complex adaptive systems and small differences in detail can lead to different impacts. This means there will always be a degree of uncertainty about the results.

Competition can be either helpful or harmful, sometimes even simultaneously depending on the objectives considered (e.g., efficiency versus equity). This implies that the instrument of competition must be used wisely and cautiously in health policy.

## Footnotes

1 An extensive literature review was organised by the European Commission to support the Expert Panel’s work. This summarising article only includes a few references. A comprehensive list of references is included in the report.

2 Competition is here defined as an instrument to encourage organisations to be more efficient and responsive to the preferences of those they serve. The role and outcomes of competition depend on context and on the availability and nature of alternative instruments. Competition provides a set of incentives that may or may not be more effective than alternatives in achieving societal goals.

3 However, Brekke et al. [2] theoretically show that, even when prices are fixed, with semi-altruistic providers and a fairly general cost structure the relationship between competition and quality is generally ambiguous. According to them, this is consistent with, and potentially explains, the mixed empirical evidence.

4 For a description of primary care and its evolution, see the Expert Opinion on primary care [7].

5 The introduction of hospital competition in the UK did not seem to worsen equity (in terms of waiting times), as had been feared, but interpretation of the findings was difficult because increased competition coincided with a major central drive to reduce waiting lists [4]. Also for the UK, Cookson et al. [3] find a negative association between market competition and elective admissions in deprived areas. The effect of pro-competition reform was to reduce this negative association slightly, suggesting that competition did not undermine equity.
